# Chronicle of Research into Lichen-Associated Bacteria

**DOI:** 10.3390/microorganisms10112111

**Published:** 2022-10-26

**Authors:** Zichen He, Takeshi Naganuma

**Affiliations:** Graduate School of Integrated Sciences for Life, Hiroshima University, 1-4-4 Kagamiyama, Higashi-Hiroshima 739-8528, Japan

**Keywords:** lichen–bacterial association, symbiosis, culture, DNA sequencing, -omics

## Abstract

Lichens are mutually symbiotic systems consisting of fungal and algal symbionts. While diverse lichen-forming fungal species are known, limited species of algae form lichens. Plasticity in the combination of fungal and algal species with different eco-physiological properties may contribute to the worldwide distribution of lichens, even in extreme habitats. Lichens have been studied systematically for more than 200 years; however, plasticity in fungal–algal/cyanobacterial symbiotic combinations is still unclear. In addition, the association between non-cyanobacterial bacteria and lichens has attracted attention in recent years. The types, diversity, and functions of lichen-associated bacteria have been studied using both culture-based and culture-independent methods. This review summarizes the history of systematic research on lichens and lichen-associated bacteria and provides insights into the current status of research in this field.

## 1. Introduction

According to one version of the story, the term “lichen” originated from the Greek “λειχήν” (leichen), meaning “licker”, and was later transcribed to “lichen” [1]. The approximately 20,000 lichen species are highly mutually symbiotic systems consisting of fungal and algal symbionts. Lichen nomenclature is based on lichen-forming fungal species. Although ~20,000 lichen-forming fungal species are known, only a few species of algae from two green algal genera, *Trebouxia* and *Cocomyxa*, form lichens. Plasticity in the combination of fungal–algal species having different eco-physiological properties may contribute to the worldwide distribution of lichens, even in maritime and extreme habitats, covering approximately 8% of Earth’s land surface [2,3,4,5]. Lichens have been studied systematically for over 200 years [6], but plasticity in fungal–algal symbiotic combinations remains unclear [7].

Lichen-like symbioses presumably developed characteristic growth styles approximately 600 million years ago [8]. Linnaeus initially classified lichens as algae [6], but this view was challenged after the discovery of “gonidia” by Wallroth in 1825 [9]. In 1867, Schwendener proposed the basic recognition of the dual association between fungi and algae [10]. Further, in 1876, Frank first proposed the concept of “symbiosis” and used it to describe lichens [11]. Moreover, in 1879, de Bary also used the concept of “symbiosis” in his book “Die Erscheinung der Symbiose” and carried this concept forward [12]. Lichens are believed to be the symbiosis of fungi and algae. Cyanobacteria were discovered as another type of photobiont closely associated with nitrogen fixation in lichens [13]. At present, approximately 20,000 lichen species have been described, most of which have algal partners as photobionts; however, only 10% of lichens are symbiotic with cyanobacteria. Approximately 2–4% of lichen-forming fungi are associated with both algal and cyanobacterial phototrophs [4,14]. Further, >2000 species of obligate lichenicolous fungi, which are not lichen-forming but lichen-parasitic, have been identified [15].

Lichens play a unique role in many spheres. For example, lichens serve as pioneer organisms in the primary succession of ecological communities [16]. They help prevent desertification and restore desert by forming a biological crust or biocrust [17,18,19]. Owing to their sensitivity to environmental changes, lichens can be used as a biological indicator to evaluate and monitor the extent of air pollution [20,21]. Further, lichens facilitate the measurement of the surface age of exposed rocks based on their growth on some exposed rocks [22]. Lichens decompose polyester resins, heavy metals, radionuclides, other pollutants, and certain pathogens; consequently, they help purify the environment [23,24,25]. Lichens serve as food for some animals in the wild [26]. Some lichen species, with beneficial health effects on humans, are used as a food source [27]. Lichens are also used to prepare dyes and perfumes [28,29]. Lichens are reportedly used in traditional medicinal preparations [30]. A study provided chemical and biological evidence for the ethnopharmacological uses of *Flavoparmelia caperata* against alcohol-induced hepatic injury [31]. Lichens produce metabolites possessing antibacterial and anti-inflammatory activities [32,33].

Biochemical and biomedical aspects are often presumed to be related to the fungal activity; however, the roles or functions of bacteria, whether symbiotic or merely associated, cannot be ruled out. This mini-review chronicles studies on lichen-associated bacteria, focusing on methodologies from classical culture-based approaches to modern culture-independent challenges, including “-omics”, with a brief mention of artificial infection.

## 2. Discovery of Other Symbiotic Components

Although lichens were initially thought to be the symbiosis of fungi and algae (or cyanobacteria), an increasing number of subsequent studies have shown that lichens are not simply binary or ternary symbionts such as bacteria, yeasts, protists, or viruses [34,35,36,37]. Therefore, some researchers refer to lichens as “holobionts” dominated by certain fungi containing a variety of microbiomes [38] and redefine lichens as a self-sufficient ecosystem formed by the interaction of a thallus-forming fungus, an extracellular arrangement of one or more photosynthetic partners, and a variable number of other microorganisms [39]. In this case, each organism has its niche in the complex lichen ecosystem and may grow on its own under certain natural or artificial conditions. Therefore, the “lichen” phenotype can be considered a symbiotic phenotype of lichen-forming fungi, which is in accordance with current research results [40]. Lichens of artificial recombinant fungal/algal partners with different properties can be generated and assessed for phenotypic features [41,42].

This review focuses on lichen-associated bacterial research in recent years. The isolation of bacteria from lichens has a century-long history. As early as 1892, Thaxter isolated the deltaproteobacterial *Chondromyces lichenicolus* (currently *Melittangium lichenicola* [43]) from lichens [44]; this may be the first bacterium to have been isolated from lichens according to existing records. In the 20th century, there was no consensus that bacteria were symbionts of lichens, but researchers successively isolated bacteria of various genera, including *Azotobacter* [45,46], *Pseudomonas* [47], *Bacillus* [47], *Beijerinckia* [48], and *Clostridium* [46] from lichens. In 1925, Uphof isolated a purple bacterium from *Chiodecton sanguineum* (now *Cryptothecia rubrocincta* [49]) and named it *Rhodobacterium lichenophora*. He also renamed *Chiodecton sanguineum* to *Rhodobacteriophora sanguinea* [50,51]. Although the relevant literature is still cited today, it should be noted that in 1926, Sucssenguth came to different conclusions in his research results and doubted the results of Uphof [52]. Grube et al. suspected a misunderstanding of secondary crystalline compounds in these two contradictory reports [53]. To date, there have been no follow-up studies or data on purple bacteria. Lichen-associated bacteria have been isolated and detected for a long time, but until the first few years of the 21st century, some researchers still referred to them as epiphytic bacteria or even bacterial contamination [54,55].

## 3. Research Methods Involving Lichen-Associated Bacteria

If contradictory reports stem from a single research method at the time, subsequent research methods combined with molecular methods considerably reduce the possibility of such contradictions. Currently, research methods for lichen-associated bacteria can be roughly divided into culture-based and culture-independent methods. Culture-based methods are used to isolate and cultivate bacteria from lichens and include various physical and chemical experimental methods to study the structure, function, products, and metabolites of bacteria. Culture-independent methods include molecular methods, such as DNA extraction, polymerase chain reaction (PCR), gel electrophoresis, and denaturing gradient gel electrophoresis (DGGE). First-generation DNA sequencing (Sanger method) and shotgun sequencing have been used to identify the isolated bacteria and perform whole-genome sequencing. Second-generation sequencing, also known as high-throughput or next-generation sequencing (NGS) (for example, 454-pyrosequencing, Illumina sequencing, and Ion Torrent sequencing), third-generation sequencing, also known as long-read sequencing (Single-Molecule Real-Time (SMRT) sequencing), and -omics technologies have only recently been used to study lichens for the identification and analysis of isolated bacteria, as well as for the compositional and functional analysis of bacterial communities. 

**Figure 1:**
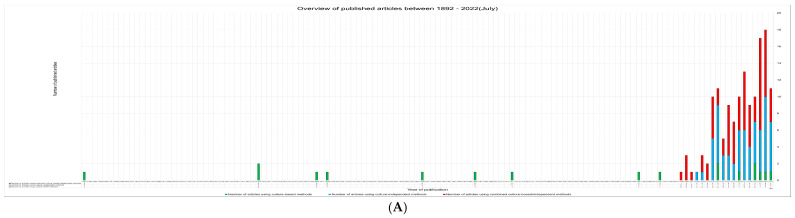

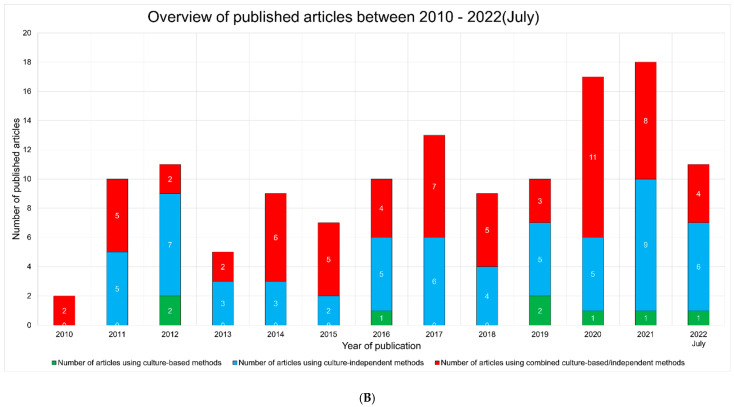
Yearly numbers of publications are listed in Table 1, as categorized by culture-based, culture-independent, and combined methods. (**A**) numbers of publications from 1892 to July 2022. (**B**) numbers of publications from 2010 to July 2022.

An extensive list of studies on lichen-associated bacteria since 1892 is shown in Table S1 and categorized based on culture-based, culture-independent, and combined methods [39,44,45,46,47,48,50,51,55,56,57,58,59,60,61,62,63,64,65,66,67,68,69,70,71,72,73,74,75,76,77,78,79,80,81,82,83,84,85,86,87,88,89,90,91,92,93,94,95,96,97,98,99,100,101,102,103,104,105,106,107,108,109,110,111,112,113,114,115,116,117,118,119,120,121,122,123,124,125,126,127,128,129,130,131,132,133,134,135,136,137,138,139,140,141,142,143,144,145,146,147,148,149,150,151,152,153,154,155,156,157,158,159,160,161,162,163,164,165,166,167,168,169,170,171,172,173,174,175,176,177,178,179,180,181,182,183,184,185,186,187,188,189,190,191,192,193,194,195,196,197]. The yearly numbers of corresponding publications are shown in Figure 1.

According to our review, the first research paper concerning the analysis of lichen-associated bacteria by culture-independent methods appeared in 2005; before that, a few existing studies on lichen-associated bacteria employed culture-based methods. In addition, in the same year, Cardinale et al. published research on the bacterial communities of several different lichens [198], which may represent the first report on bacterial communities, and published a related paper in 2006 [34]. In 2006, a study by Liba et al. first used Sanger DNA sequencing to analyze isolated nitrogen-fixing bacteria on a large scale [193], assuming that bacteria exist as symbionts. In 2008, Cardinale et al. pioneered the use of FISH to analyze bacterial communities in lichens, which was the first study to use only culture-independent methods [191]. In 2011, Mushegian et al. used second-generation sequencing methods (454-pyrosequencing) combined with Terminal Restriction Fragment Length Polymorphism analysis (T-RFLP) to analyze lichen bacterial communities [179]. In the same year, Schneider et al. pioneered the use of meta-proteomics to analyze the function of lichen-associated bacteria, which was the first to rely on culture-independent methods to analyze the function of lichen-associated bacteria [178]. In subsequently published papers, many researchers have used a variety of culture-independent methods to analyze the functions of lichen-associated bacteria; however, the culture-independent methods chosen by most researchers were similar to those described above. 

After discovering the suitability of culture-independent methods for studying lichen-associated bacteria, most studies gradually selected new techniques for culture-independent methods. Among the associated papers referenced in this review, 88.1% used culture-independent methods to study lichen-associated bacteria, which is close to 95% considering the emergence and generalization time of the technology. Perhaps, considering convenience and accuracy, many researchers have abandoned culture-based methods and devoted themselves to culture-independent research. Papers that reported only culture-based or culture-independent methods accounted for 11.9% and 41.1% of the total, respectively. Papers that used both methods accounted for 47.0% of the total. A taxonomic summary of publications using culture-based, culture-independent, and combined methods is presented in Table 1.

Lichen species and lichen-associated bacteria studied since 1892 are shown in Table S1. Lichen species were as accurate as possible at the species level. To facilitate the statistics of the types of related bacteria, this review classifies all bacteria studied using culture-based and culture-independent methods at the phylum level, and the phylum names are based on the latest validation [199]. A total of 145 of 151 publications reported isolation or analysis of specific species of lichen-associated bacteria using culture-based or culture-independent methods or both. Several interesting results were obtained. Among them, eleven publications reported culture-based methods for isolating *Actinomycetota*, probably because their functions have attracted the interest of researchers. Strains of *Pseudomonadota* were isolated and analyzed, as indicated in two publications, using both culture-based and culture-independent methods. *Pseudomonadota* was identified in 69.5% of all the publications and regarded as the dominant phylum. Of the 103 publications that isolated or detected *Pseudomonadota*, almost half used culture-based and culture-independent methods. The second most dominant phylum was *Actinomycetota*, accounting for 40.7% of all the publications. The phyla *Bacteroidota* and *Bacillota* were identified using both culture-based/independent methods, whereas *Acidobacteriota* was identified only using culture-independent methods. *Cyanobacteria* was mostly identified using metagenomics or in some publications without deletion of related sequences. *Gemmatimonadetes* and *Planctomycetota* were reported as dominant phyla once in two different publications in 2020 and 2012, respectively.

## 4. Problems and Challenges

Culture-independent molecular methods, such as high-throughput sequencing tools, reveal the species diversity of unexplored microbial communities and reveal the presence of many novel microorganisms previously undetectable by culture-based methods. These methods rely on culture-independent methods to directly analyze the function of lichen-associated bacteria. Culture-based methods are critical for the discovery of useful bioactive compounds. Combined with molecular techniques, the identification of isolated strains and analysis of metabolites have been greatly facilitated, with a reduced possibility of errors. In addition, some researchers insist on using only culture-based methods to analyze bacteria.

However, it is worth noting that methodological variety has, rather unfortunately, resulted in some drawbacks due to methodological non-uniformity. For example, different media used in culture-based methods have isolated different strains, leading to different results when evaluating the numbers and diversity of lichen-associated bacteria. Therefore, some researchers have used various media simultaneously to lessen the impact of this issue. In addition, some researchers have used both culture-based and culture-independent molecular methods to evaluate the number and diversity of lichen-associated bacteria. Pankratov et al. pointed out that no correlations are seen between the cultured bacteria and molecular genetic information of lichen-associated bacteria [200].

A major challenge in lichen-associated microbiology is the prediction of the roles or functions of bacteria in lichens. Pankratov et al. concluded that the most obvious functions of the bacterial communities in lichens are: (1) nitrogen fixation; (2) production of secondary metabolites, such as growth regulators, vitamins, antibiotics, ethylene, and indole acetic acid, as well as the production of “lichenic acid”; (3) degradation of thallus and migration of macro and trace elements to lichen growth sites; (4) formation of carbohydrate pools as polysaccharides of bacterial origin [200]. Studies concerning the functions of lichen-associated bacteria are listed in Table S1 by publications dating back to 1892, including all data that can be found, which is essentially consistent with the above-mentioned summary [39,44,45,46,47,48,50,51,55,56,57,58,59,60,61,62,63,64,65,66,67,68,69,70,71,72,73,74,75,76,77,78,79,80,81,82,83,84,85,86,87,88,89,90,91,92,93,94,95,96,97,98,99,100,101,102,103,104,105,106,107,108,109,110,111,112,113,114,115,116,117,118,119,120,121,122,123,124,125,126,127,128,129,130,131,132,133,134,135,136,137,138,139,140,141,142,143,144,145,146,147,148,149,150,151,152,153,154,155,156,157,158,159,160,161,162,163,164,165,166,167,168,169,170,171,172,173,174,175,176,177,178,179,180,181,182,183,184,185,186,187,188,189,190,191,192,193,194,195,196,197]. Many researchers have been interested in the functions of *Actinomycetota*. None of these functions is unique to lichens; bacteria also exhibit some of these functions, whether they are symbiotic/associated with other organisms or live alone. Most importantly, there have been no complete experiments or evidence to determine the roles of bacteria in lichens, and researchers usually make assumptions based on the featured functions of isolated bacteria or known functions of relevant taxa. Increasing the accuracy of such assumptions is an urgent issue, particularly when employing culture-independent methods and omics or meta-omics.

An unprecedented challenge in lichenology is the artificial infection of a selected bacterium or a set of selected bacteria to pre-sterilized lichens. Agarwood (*Aquilaria malaccensis*) exemplifies artificial inoculation. Its endophytic fungal/bacterial isolates are inoculated to enhance the production of natural incense [201]. Agarwood surface is pre-sterilized with ethanol and sodium hypochlorite [202]. Lichens are also surface-sterilized with ethanol, sodium hypochlorite, or hydrogen peroxide [203,204], but artificial infection against lichens has still been an intriguing challenge.

## 5. Conclusions

Lichens have been systematically studied for over 200 years, but their nature remains poorly understood, particularly plasticity in fungal–algal symbiotic combinations. As to bacterial associates of lichens, traditional culture-based methods reveal their physiological and biochemical features in vitro but allow only inferences on their roles in vivo in lichens. Culture-independent molecular approaches provide taxonomic and phylogenetic identifications of bacterial associates but only in silico speculations on their functions in lichens. Considering the variety and practicality of secondary metabolites produced by lichen-forming fungi and bacteria, lichen-associated bacteria would represent huge treasure houses for human benefit. However, research on lichen-associated bacteria is still limited. For research methods targeting lichen-associated bacteria, the publications found and summarized in this review do not necessarily cover all, and related studies are urgently needed to develop a more complete and accurate understanding. Overall, much remains to be accomplished in advancing research on lichen-associated bacteria.

## Figures and Tables

**Table 1:** A list of 151 publications on the isolation or analysis of lichen-associated bacteria, categorized based on culture-based, culture-independent, and combined methods. Detected major phyla are listed. The numbers of all relevant publications and publications using culture-based, culture-independent, and combined methods are shown.

Phylum	Total Number of Publications	Number of Publications Using Culture-Based Methods	Number of Publications Using Culture-Independent Methods	Number of Publications Using Combined Culture-Based/Independent Methods
*Pseudomonadota*	103	50	51	2
*Actinomycetota*	59	40	19	0
*Bacteroidota*	27	13	14	0
*Acidobacteriota*	25	0	25	0
*Bacillota*	21	15	6	0
*Cyanobacteria*	8	0	8	0
*Gemmatimonadetes*	1	0	1	0
*Planctomycetota*	1	0	1	0

## Supplementary Materials

The following supporting information can be downloaded at: https://www.mdpi.com/article/10.3390/microorganisms10112111/s1, Table S1: Total of 151 original articles on lichen-associated bacteria research since 1892 to 2022 (July) by descending order, including 145 original papers, 4 theses and 2 reports. The subjects, methods, information of lichen samples, phyla and analyzed functions of isolated bacteria based on different methods are listed in the table are only those-associated to bacteria respectively

## References

[1] Beekes R.S.P., Lubotsky A. (2009). Etymological Dictionary of Greek. Leiden Indo-European Etymological Dictionary Series.

[2] Nash I.I.I., Thomas H. (2008). Lichen Biology.

[3] Allen J.L., Lendemer J.C. (2022). A call to reconceptualize lichen symbioses. Trends Ecol. Evol..

[4] Grimm M., Grube M., Schiefelbein U., Zühlke D., Bernhardt J., Riedel K. (2021). The lichens’ microbiota, still a mystery?. Front. Microbiol..

[5] Harada H. (2021). Illustrated flora of marine and maritime lichens of Japan (4), *Pyrenopsis conturvatula* (*Lichinaceae*). Lichenology.

[6] Linnaeus C. (1753). Species Plantarum.

[7] Sanders W., Masumoto H. (2021). Lichen algae: The photosynthetic partners in lichen symbioses. Lichenologist.

[8] Yuan X., Xiao S., Taylor T.N. (2005). Lichen-like symbiosis 600 million years ago. Science.

[9] Pitt C.C. (1919). A short history of lichenology. Bryologist.

[10] (1867). Protokoll der botanischen Sektion. Verh. Schweiz. Naturf. Ges..

[11] Frank A.B. (1876). Ueber die biologischen: Verhältnisse des Thollus einiger Krustenflechten. Flora Allg. Bot. Ztg..

[12] De Bary A. (1879). Die Erscheinung der Symbiose.

[13] Peters G.A., Toia R.E., Calvert H.E., Marsh B.H., Skinner F.A., Uomala P. (1986). Lichens to Gunnera—With emphasis on Azolla. Nitrogen Fixation with Non-Legumes: The Third International Symposium on Nitrogen Fixation with Non-Legumes, Helsinki, 2–8 September 1984.

[14] Lücking R., Hodkinson B.P., Leavitt S.D. (2017). The 2016 classification of lichenized fungi in the Ascomycota and Basidiomycota—Approaching one thousand genera. Bryologist.

[15] Diederich P., Lawrey J.D., Ertz D. (2018). The 2018 classification and checklist of lichenicolous fungi, with 2000 non-lichenized, obligately lichenicolous taxa. Bryologist.

[16] Gilbert O.L. (1990). The Lichen Flora of Urban Wasteland. Lichenologist.

[17] Bowker M.A., Belnap J., Davidson D.W., Phillips S.L. (2005). Evidence for micronutrient limitation of biological soil crusts: Importance to arid-lands restoration. Ecol. Appl..

[18] Ballesteros M., Ayerbe J., Casares M., Cañadas E.M., Lorite J. (2017). Successful lichen translocation on disturbed gypsum areas: A test with adhesives to promote the recovery of biological soil crusts. Sci. Rep..

[19] Finger-Higgens R., Duniway M.C., Fick S., Geiger E.L., Hoover D.L., Pfennigwerth A.A., Van Scoyoc M.W., Belnap J. (2022). Decline in biological soil crust N-fixing lichens linked to increasing summertime temperatures. Proc. Natl. Acad. Sci. USA.

[20] Rose C.I., Hawksworth D.L. (1981). Lichen recolonization in London’s cleaner air. Nature.

[21] Hawksworth D.L., Rose F. (1979). Lichens as Pollution Monitors.

[22] Innes J.L. (1985). Lichenometry. Prog. Phys. Geogr..

[23] Cappitelli F., Sorlini C. (2008). Microorganisms attack synthetic polymers in items representing our cultural heritage. Appl. Environ. Microbiol..

[24] Gadd G.M. (2010). Metals, minerals and microbes: Geomicrobiology and bioremediation. Microbiology.

[25] Johnson C.J., Bennett J.P., Biro S.M., Duque-Velasquez J.C., Rodriguez C.M., Bessen R.A., Rocke T.E. (2011). Degradation of the disease-associated prion protein by a serine protease from lichens. PLoS ONE.

[26] Skogland T. (1984). Wild reindeer foraging-niche organization. Ecography.

[27] Zhao Y., Wang M., Xu B. (2021). A comprehensive review on secondary metabolites and health-promoting effects of edible lichen. J. Funct. Foods.

[28] Calà E., Benzi M., Gosetti F., Zanin A., Gulmini M., Idone A., Serafini I., Ciccola A., Curini R., Whitworth I. (2019). Towards the identification of the lichen species in historical orchil dyes by HPLC-MS/MS. Microchem. J..

[29] Joulain D., Tabacchi R. (2009). Lichen extracts as raw materials in perfumery. Part 1: Oakmoss. Flavour Fragr. J..

[30] Crawford S.D., Ranković B. (2019). Lichens used in traditional medicine. Lichen Secondary Metabolites: Bioactive Properties and Pharmaceutical Potential.

[31] Shukla I., Azmi L., Rao C.V., Jawaid T., Kamal M., Alkhamees O.A., Alaseem A.M., Alsanad S.M. (2022). Inclusive roles of protocetraric acid, a secondary metabolite from the common green shield lichen *Flavoparmelia caperata* in alcohol-induced hepatic injury. Lat. Am. J. Pharm..

[32] Müller K. (2001). Pharmaceutically relevant metabolites from lichens. Appl. Microbiol. Biotechnol..

[33] Ranković B., Mišić M., Sukdolak S. (2008). The antimicrobial activity of substances derived from the lichens *Physcia aipolia*, *Umbilicaria polyphylla*, *Parmelia caperata* and *Hypogymnia physodes*. World J. Microbiol. Biotechnol..

[34] Cardinale M., Puglia A.M., Grube M. (2006). Molecular analysis of lichen-associated bacterial communities. FEMS Microbiol. Ecol..

[35] Spribille T., Tuovinen V., Resl P., Vanderpool D., Wolinski H., Aime M.C., Schneider K., Stabentheiner E., Toome-Heller M., Thor G. (2016). Basidiomycete yeasts in the cortex of ascomycete macrolichens. Science.

[36] Wilkinson D.M., Creevy A.L., Kalu C.L., Schwartzman D.W. (2015). Are heterotrophic and silica-rich eukaryotic microbes an important part of the lichen symbiosis?. Mycology.

[37] Petrzik K., Koloniuk I., Sehadová H., Sarkisova T. (2019). Chrysoviruses inhabited symbiotic fungi of lichens. Viruses.

[38] Simon J.-C., Marchesi J.R., Mougel C., Selosse M.-A. (2019). Host-microbiota interactions: From holobiont theory to analysis. Microbiome.

[39] Hawksworth D.L., Grube M. (2020). Lichens redefined as complex ecosystems. New Phytol..

[40] Honegger R., Hock B. (2012). 15 The Symbiotic phenotype of lichen-forming ascomycetes and their endo- and epibionts. Fungal Associations.

[41] Williams L., Colesie C., Ullmann A., Westberg M., Wedin M., Büdel B. (2017). Lichen acclimation to changing environments: Photobiont switching vs. climate-specific uniqueness in *Psora decipiens*. Ecol. Evol..

[42] Zakeri Z., Junne S., Jäger F., Dostert M., Otte V., Neubauer P. (2022). Lichen cell factories: Methods for the isolation of photobiont and mycobiont partners for defined pure and co-cultivation. Microb. Cell Fact..

[43] McCurdy H.D. (1971). Studies on the taxonomy of the Myxobacterales: IV. Melittangium. Int. J. Syst. Evol. Microbiol..

[44] Thaxter R. (1892). On the Myxobacteriaceæ, a new order of Schizomycetes. Bot. Gaz..

[45] Genkel P.A., Yuzhakova L.A. (1936). Nitrogen-fixing bacteria in lichens. Proc. Perm. Biol. Res. Inst..

[46] Iskina R. (1938). On nitrogen fixing bacteria in lichens. Isv. Biol. Inst. Permsk..

[47] Panosyan A., Nikogosyan V. (1966). The presence of *Azotobacter* in lichens. Akad. Nauk. Armian. SSR Biol. Zhurn. Armen..

[48] Genkel P.A., Plotnikova T.T. (1973). Nitrogen-fixing bacteria in lichens. Izv. Akad. Nauk. SSSR Biol..

[49] Thor G. (1991). The Placement of *Chiodecton sanguineum* (syn. Chiodecton rubrocinctum), and Cryptothecia striata sp. nov. Bryologist.

[50] Uphof J.C.T. (1925). Purple bacteria as symbionts of a lichen. Science.

[51] Uphof J.C.T. (1925). The occurrence of purple bacteria as symbionts of a lichen. Am. J. Bot..

[52] Suessenguth K. (1926). Zur frage der vergesellschaftung von flechten mit purpurbakterien. Ber. Deutsch. Bot. Ges..

[53] Grube M., Berg G. (2009). Microbial consortia of bacteria and fungi with focus on the lichen symbiosis. Fungal Biol. Rev..

[54] Díaz E.-M., Rodríguez S., Quintana J. (2008). Epiphytic Bacteria on Lichens.

[55] Schieleit P., Ott S. (1997). Ethylene production in lichens with respect to possible bacterial contamination. Lichenologist.

[56] Xu H., Wang L., Feng X., Gong X. (2022). Core taxa and photobiont-microbial interaction within the lichen *Heterodermia obscurata* (*Physcsiaceae*, *Heterodermia*). Symbiosis.

[57] Subbaiyan R., Ganesan A., Ramasubramanian B. (2022). Self-potent anti-microbial and anti-fouling action of silver nanoparticles derived from lichen-associated bacteria. Appl. Nanosci..

[58] Pankratov T.A., Nikitin P.A., Patutina E.O. (2022). Genome analysis of two lichen bacteriobionts, *Lichenibacterium ramalinae* and *Lichenibacterium minor*: Toxin–antitoxin systems and secretion proteins. Microbiology.

[59] Rolshausen G., Dal Grande F., Otte J., Schmitt I. (2022). Lichen holobionts show compositional structure along elevation. Mol. Ecol..

[60] Alonso-García M., Villarreal J.C. (2022). Bacterial community of reindeer lichens differs between northern and southern lichen woodlands. Can. J. For. Res..

[61] Vijayakumar V.R., Saravanan K., Somasundaram M., Jayaraj R., Annamalai P., Nooruddin T., Dharumadurai D. (2022). Meta-genomic analysis of lichen-associated bacterial community profiling in *Roccella montagnei*. Arch. Microbiol..

[62] Somphong A., Poengsungnoen V., Buaruang K., Suriyachadkun C., Sripreechasak P., Tanasupawat S., Phongsopitanun W. (2022). Diversity of the culturable lichen-derived actinobacteria and the taxonomy of *Streptomyces parmotrematis* sp. nov. Antonie Leeuwenhoek.

[63] Ghimire N., Kim B., Lee C.-M., Oh T.-J. (2022). Comparative genome analysis among *Variovorax* species and genome guided aromatic compound degradation analysis emphasizing 4-hydroxybenzoate degradation in *Variovorax* sp. PAMC26660. BMC Genom..

[64] Park Y., Noh H.-J., Hwang C.Y., Shin S.C., Hong S.G., Jin Y.K., Lee H., Lee Y.M. (2022). *Hymenobacter siberiensis* sp. nov., isolated from a marine sediment of the East Siberian Sea and *Hymenobacter psoromatis* sp. nov., isolated from an Antarctic lichen. Int. J. Syst. Evol. Microbiol..

[65] Choi E., Huh A., Oh C., Oh J.-I., Kang H.Y., Hwang J. (2022). Functional characterization of HigBA toxin-antitoxin system in an Arctic bacterium, *Bosea* sp. PAMC 26642. J. Microbiol..

[66] Gupta S., Han S.-R., Kim B., Lee C.-M., Oh T.-J. (2022). Comparative analysis of genome-based CAZyme cassette in Antarctic *Microbacterium* sp. PAMC28756 with 31 other *Microbacterium* species. Genes Genom..

[67] Noël A., Garnier A., Clément M., Rouaud I., Sauvager A., Bousarghin L., Vásquez-Ocmín P., Maciuk A., Tomasi S. (2021). Lichen-associated bacteria transform antibacterial usnic acid to products of lower antibiotic activity. Phytochemistry.

[68] Noh H.-J., Park Y., Hong S.G., Lee Y.M. (2021). Diversity and physiological characteristics of Antarctic lichens-associated bacteria. Microorganisms.

[69] Swamy C.T., Gayathri D. (2021). High throughput sequencing study of foliose lichen-associated bacterial communities from India. Mol. Biol. Rep..

[70] Leiva D., Fernández-Mendoza F., Acevedo J., Carú M., Grube M., Orlando J. (2021). The bacterial community of the foliose macro-lichen *Peltigera frigida* is more than a mere extension of the microbiota of the subjacent substrate. Microb. Ecol..

[71] Zhang Y., Wu F., Su M., He D., Gu J.-D., Guo Q., Kakakhel M.A., Yang Y., Wang W., Feng H. (2021). Spatial and temporal distributions of microbial diversity under natural conditions on the sandstone stelae of the Beishiku Temple in China. Int. Biodeterior. Biodegrad..

[72] Hei Y., Zhang H., Tan N., Zhou Y., Wei X., Hu C., Liu Y., Wang L., Qi J., Gao J.-M. (2021). Antimicrobial activity and biosynthetic potential of cultivable actinomycetes associated with lichen symbiosis from Qinghai-Tibet Plateau. Microbiol. Res..

[73] Shishido T.K., Wahlsten M., Laine P., Rikkinen J., Lundell T., Auvinen P. (2021). Microbial communities of *Cladonia* lichens and their biosynthetic gene clusters potentially encoding natural products. Microorganisms.

[74] Da Silva A.V., de Oliveira A.J., Tanabe I.S.B., Silva J.V., da Silva Barros T.W., da Silva M.K., França P.H.B., Leite J., Putzke J., Montone R. (2021). Antarctic lichens as a source of phosphate-solubilizing bacteria. Extremophiles.

[75] Lang L., An D.-F., Jiang L.-Q., Li G.-D., Wang L.-S., Wang X.-Y., Li Q.-Y., Jiang C.-L., Jiang Y. (2021). *Paracoccus lichenicola* sp. nov., isolated from lichen. Curr. Microbiol..

[76] Grzesiak J., Woltyńska A., Zdanowski M.K., Górniak D., Świątecki A., Olech M.A., Aleksandrzak-Piekarczyk T. (2021). Metabolic fingerprinting of the Antarctic cyanolichen *Leptogium puberulum*–associated bacterial community (Western Shore of Admiralty Bay, King George Island, Maritime Antarctica). Microb. Ecol..

[77] Alonso-García M., Villarreal J.C. (2021). Geography, not host identity, shapes bacterial community in reindeer lichens. bioRxiv.

[78] Shrestha P., Han S.-R., Lee J.H., Park H., Oh T.-J. (2021). A computational approach to identify CRISPR-Cas loci in the complete genomes of the lichen-associated *Burkholderia* sp. PAMC28687 and PAMC26561. Genomics.

[79] Wicaksono W.A., Kusstatscher P., Erschen S., Reisenhofer-Graber T., Grube M., Cernava T., Berg G. (2021). Antimicrobial-specific response from resistance gene carriers studied in a natural, highly diverse microbiome. Microbiome.

[80] Ghimire N., Han S.-R., Kim B., Jung S.-H., Park H., Lee J.H., Oh T.-J. (2021). Complete genome sequencing and comparative CAZyme analysis of *Rhodococcus* sp. PAMC28705 and PAMC28707 provide insight into their biotechnological and phytopathogenic potential. Arch. Microbiol..

[81] Shrestha P., Han S.-R., Lee J.H., Park H., Oh T.-J. (2021). Comparative genome analysis of carbohydrate-active enzymes and virulence factors in lichen-associated *Variovorax* sp. PAMC28711. Preprint.

[82] Ullah J., Khanum Z., Khan I.A., Khalid A.N., Musharraf S.G., Ali A. (2021). Metaproteomics reveals the structural and functional diversity of *Dermatocarpon miniatum* (L.) *W. Mann.* Microbiota. Fungal Biol..

[83] VidhyaSri A.R., ThamaraiSelvi B., Sanjay Prasad S., Karkuvelraja R. (2021). Isolation of Lichens associated Actinomycetes: Determining its antibacterial activity against Multi drug resistant *Klebsiella pneumoniae* and Methicillin resistant *Staphylococcus aureus*. J. Univ. Shanghai Sci. Technol..

[84] An D.-F., Jiang L.-Q., Zhang K., Li G.-D., Wang X.-Y., Jiang M.-G., Lang L., Wang L.-S., Imhoff J.F., Jiang C.-L. (2021). *Glaciibacter flavus* sp. nov., isolated from a lichen sample. Arch. Microbiol..

[85] Tran K.N., Pham N., Jang S.-H., Lee C. (2020). Purification and characterization of a novel medium-chain ribitol dehydrogenase from a lichen-associated bacterium *Sphingomonas* sp.. PLoS ONE.

[86] Sierra M.A., Danko D.C., Sandoval T.A., Pishchany G., Moncada B., Kolter R., Mason C.E., Zambrano M.M. (2020). The Microbiomes of Seven Lichen Genera Reveal Host Specificity, a Reduced Core Community and Potential as Source of Antimicrobials. Front. Microbiol..

[87] Rajaram S.K., Ahmad P., Sujani Sathya Keerthana S., Jeya Cressida P., Ganesh Moorthy I., Suresh R.S.S. (2020). Extraction and purification of an antimicrobial bioactive element from lichen associated *Streptomyces olivaceus* LEP7 against wound inhabiting microbial pathogens. J. King Saud Univ. Sci..

[88] Greshake Tzovaras B., Segers F.H.I.D., Bicker A., Dal Grande F., Otte J., Anvar S.Y., Hankeln T., Schmitt I., Ebersberger I. (2020). What Is in *Umbilicaria pustulata*? A Metagenomic Approach to Reconstruct the Holo-Genome of a Lichen. Genome Biol. Evol..

[89] Puvar A.C., Nathani N.M., Shaikh I., Bhatt A.D., Bhargava P., Joshi C.G., Joshi M.N. (2020). Bacterial line of defense in *Dirinaria* lichen from two different ecosystems: First genomic insights of its mycobiont *Dirinaria* sp. GBRC AP01. Microbiol. Res..

[90] Ghimire N., Han S.-R., Kim B., Park H., Lee J.H., Oh T.-J. (2020). Comparative genomic study of polar lichen-associated *Hymenobacter* sp. PAMC 26554 and *Hymenobacter* sp. PAMC 26628 reveals the presence of polysaccharide-degrading ability based on habitat. Curr. Microbiol..

[91] Noh H.-J., Shin S.C., Park Y., Choi A., Baek K., Hong S.G., Cho Y.-J., Lee H., Lee Y.M. (2020). *Lichenicola cladoniae* gen. nov., sp. nov., a member of the family *Acetobacteraceae* isolated from an Antarctic lichen. Int. J. Syst. Evol. Microbiol..

[92] Wicaksono W.A., Cernava T., Grube M., Berg G., Stewart F.J. (2020). Assembly of bacterial genomes from the metagenomes of three lichen species. Microbiol. Resour. Announc..

[93] Klarenberg I.J., Keuschnig C., Warshan D., Jónsdóttir I.S., Vilhelmsson O. (2020). The total and active bacterial community of the chlorolichen *Cetraria islandica* and its response to long-term warming in sub-Arctic tundra. Front. Microbiol..

[94] Liu C., Jiang Y., Huang R., Jiang B., Zheng K., Wu S. (2020). Diverse secondary metabolites from a lichen-derived *Amycolatopsis* strain. Curr. Microbiol..

[95] Noh H.J., Lee Y.M., Park C.H., Lee H.K., Cho J.C., Hong S.G. (2020). Microbiome in *Cladonia squamosa* is vertically stratified according to microclimatic conditions. Front. Microbiol..

[96] Vobis G., Solans M., Scervino J.M., Schumann P., Spröer C., Messuti M.I. (2020). Isolation and characterization of an endolichenic actinobacterium from the lichen thallus of *Pseudocyphellaria berberina*. Symbiosis.

[97] Jin Y., Aobulikasimu N., Zhang Z., Liu C., Cao B., Lin B., Guan P., Mu Y., Jiang Y., Han L. (2020). Amycolasporins and dibenzoyls from lichen-associated *Amycolatopsis hippodromi* and their antibacterial and anti-inflammatory activities. J. Nat. Prod..

[98] Zhang K., Jiang L.-Q., Wang L.-S., An D.-F., Lang L., Li G.-D., Wang X.-Y., Shi S.-B., Li Q.-Y., Jiang C.-L. (2020). *Aureimonas leprariae* sp. nov., isolated from a *Lepraria* sp. lichen. Curr. Microbiol..

[99] Dawoud T.M., Alharbi N.S., Theruvinthalakal A.M., Thekkangil A., Kadaikunnan S., Khaled J.M., Almanaa T.N., Sankar K., Innasimuthu G.M., Alanzi K.F. (2020). Characterization and antifungal activity of the yellow pigment produced by a *Bacillus* sp. DBS4 isolated from the lichen *Dirinaria agealita*. Saudi J. Biol. Sci..

[100] Pankratov T.A., Grouzdev D.S., Patutina E.O., Kolganova T.V., Berestovskaya J.J., Ashikhmin A.A. (2020). *Lichenicoccus roseus* gen. nov., sp. nov., the first bacteriochlorophyll a-containing, psychrophilic and acidophilic *Acetobacteraceae* bacteriobiont of lichen *Cladonia* species. Int. J. Syst. Evol. Microbiol..

[101] Pankratov T.A., Grouzdev D.S., Patutina E.O., Kolganova T.V., Suzina N.E., Berestovskaya J.J. (2020). *Lichenibacterium ramalinae* gen. nov, sp. nov., *Lichenibacterium minor* sp. nov., the first endophytic, *beta*-carotene producing bacterial representatives from lichen thalli and the proposal of the new family *Lichenibacteriaceae* within the order *Rhizobiales*. Antonie Leeuwenhoek.

[102] Nahar S., Jeong M.-H., Hur J.-S. (2019). Lichen-associated bacterium, a novel bioresource of polyhydroxyalkanoate (PHA) production and simultaneous degradation of naphthalene and anthracene. J. Microbiol. Biotechnol..

[103] Cernava T., Aschenbrenner I.A., Soh J., Sensen C.W., Grube M., Berg G. (2019). Plasticity of a holobiont: Desiccation induces fasting-like metabolism within the lichen microbiota. ISME J..

[104] Fernández-Brime S., Muggia L., Maier S., Grube M., Wedin M. (2019). Bacterial communities in an optional lichen symbiosis are determined by substrate, not algal photobionts. FEMS Microbiol. Ecol..

[105] Noh H.-J., Baek K., Hwang C.Y., Shin S.C., Hong S.G., Lee Y.M. (2019). *Lichenihabitans psoromatis* gen. nov., sp. nov., a member of a novel lineage (*Lichenihabitantaceae* fam. nov.) within the order of *Rhizobiales* isolated from Antarctic lichen. Int. J. Syst. Evol. Microbiol..

[106] Zheng K.-X., Jiang Y., Jiang J.-X., Huang R., He J., Wu S.-H. (2019). A new phthalazinone derivative and a new isoflavonoid glycoside from lichen-associated *Amycolatopsis* sp.. Fitoterapia.

[107] Moreira-Grez B., Tam K., Cross A.T., Yong J.W.H., Kumaresan D., Nevill P., Farrell M., Whiteley A.S. (2019). The bacterial microbiome associated with arid biocrusts and the biogeochemical influence of biocrusts upon the underlying soil. Front. Microbiol..

[108] Kim B., Han S.-R., Lamichhane J., Park H., Oh T.-J. (2019). Draft genome analysis of antimicrobial *Streptomyces* isolated from Himalayan lichen. J. Microbiol. Biotechnol..

[109] Tzovaras B.G., Segers F.H.I.D., Bicker A., Grande F.D., Otte J., Anvar S.Y., Hankeln T., Schmitt I., Ebersberger I. (2019). What is in a lichen? A metagenomic approach to reconstruct the holo-genome of *Umbilicaria pustulata*. bioRxiv.

[110] Pham N., Pham K., Lee C., Jang S.-H. (2019). Novel insight into the role of thiamine for the growth of a lichen-associated Arctic bacterium, *Sphingomonas* sp., in the light. Korean J. Microbiol..

[111] Han S.-R., Kim D.W., Kim B., Chi Y.M., Kang S., Park H., Jung S.-H., Lee J.H., Oh T.-J. (2019). Complete genome sequencing of *Shigella* sp. PAMC 28760: Identification of CAZyme genes and analysis of their potential role in glycogen metabolism for cold survival adaptation. Microb. Pathog..

[112] Sanchez-Hidalgo M., González I., Diaz-Munoz C., Martínez G., Genilloud O. (2018). Comparative genomics and biosynthetic potential analysis of two lichen-isolated *Amycolatopsis* strains. Front. Microbiol..

[113] Almendras K., García J., Carú M., Orlando J. (2018). Nitrogen-fixing bacteria associated with *Peltigera* cyanolichens and *Cladonia* chlorolichens. Molecules.

[114] West N.J., Parrot D., Fayet C., Grube M., Tomasi S., Suzuki M.T. (2018). Marine cyanolichens from different littoral zones are associated with distinct bacterial communities. PeerJ.

[115] Parrot D., Intertaglia L., Jehan P., Grube M., Suzuki M.T., Tomasi S. (2018). Chemical analysis of the Alphaproteobacterium strain MOLA1416 associated with the marine lichen *Lichina pygmaea*. Phytochemistry.

[116] Cernava T., Vasfiu Q., Erlacher A., Aschenbrenner I.A., Francesconi K., Grube M., Berg G. (2018). Adaptions of lichen microbiota functioning under persistent exposure to arsenic contamination. Front. Microbiol..

[117] Ma J., Cao B., Liu C., Guan P., Mu Y., Jiang Y., Han L., Huang X. (2018). Actinofuranones D-I from a lichen-associated Actinomycetes, *Streptomyces gramineus*, and their anti-inflammatory effects. Molecules.

[118] Honegger R., Blanz P. (2018). Fossil lichens from the Lower Devonian and their bacterial and fungal epi-and endobionts. Biodiversity and Ecology of Fungi, Lichens and Mosses. Kerner von Marilaun Workshop 2015 in Memory of Josef Poelt.

[119] Graham L.E., Trest M.T., Will-Wolf S., Miicke N.S., Atonio L.M., Piotrowski M.J., Knack J.J. (2018). Microscopic and Metagenomic Analyses of *Peltigera ponojensis* (Peltigerales, Ascomycota). Int. J. Plant Sci..

[120] Nguyen T.B.L. (2018). Discovery of Active Secondary Metabolites from *Paenibacillus odorifer*, a Lichen-Associated Bacterium. Ph.D. Thesis.

[121] Cernava T., Erlacher A., Aschenbrenner I.A., Krug L., Lassek C., Riedel K., Grube M., Berg G. (2017). Deciphering functional diversification within the lichen microbiota by meta-omics. Microbiome.

[122] Liu C., Jiang Y., Wang X., Chen D., Chen X., Wang L., Han L., Huang X., Jiang C. (2017). Diversity, antimicrobial activity, and biosynthetic potential of cultivable actinomycetes associated with lichen symbiosis. Microb. Ecol..

[123] Noël A., Ferron S., Rouaud I., Gouault N., Hurvois J.-P., Tomasi S. (2017). Isolation and structure Identification of novel brominated diketopiperazines from *Nocardia ignorata*—A lichen-associated actinobacterium. Molecules.

[124] Eymann C., Lassek C., Wegner U., Bernhardt J., Fritsch O.A., Fuchs S., Otto A., Albrecht D., Schiefelbein U., Cernava T. (2017). Symbiotic interplay of fungi, algae, and bacteria within the lung lichen *Lobaria pulmonaria* L. Hoffm. as assessed by state-of-the-art metaproteomics. J. Proteome Res..

[125] Aschenbrenner I.A., Cernava T., Erlacher A., Berg G., Grube M. (2017). Differential sharing and distinct co-occurrence networks among spatially close bacterial microbiota of bark, mosses and lichens. Mol. Ecol..

[126] Kono M., Tanabe H., Ohmura Y., Satta Y., Terai Y. (2017). Physical contact and carbon transfer between a lichen-forming *Trebouxia* alga and a novel *Alphaproteobacterium*. Microbiology.

[127] Si H.-L., Shi F.-X., Zhang L.-L., Yue H.-S., Wang H.-Y., Zhao Z.-T. (2017). *Subtercola lobariae* sp. nov., an actinobacterium of the family *Microbacteriaceae* isolated from the lichen *Lobaria retigera*. Int. J. Syst. Evol. Microbiol..

[128] Kim J., Kwon K.K., Kim B.K., Hong S.G., Oh H.-M. (2017). Genome sequence of *Caballeronia sordidicola* strain PAMC 26592 isolated from an arctic lichen species. Korean J. Microbiol..

[129] Yang J.A., Hong S.G., Oh H.-M. (2017). Genome sequence of *Caballeronia sordidicola* strain PAMC 26577 isolated from *Cladonia* sp., an Arctic lichen species. Korean J. Microbiol..

[130] Figàs Segura À. (2017). Bacterial Communities Associated with the Lichen Ramalina farinacea (L.) Ach.: Composition, Biodiversity and Biotechnological Potential. Doctoral Thesis.

[131] Liu C., Jiang Y., Lei H., Chen X., Ma Q., Han L., Huang X. (2017). Four new nanaomycins produced by *Streptomyces hebeiensis* derived from lichen. Chem. Biodivers..

[132] Kim J., Kwon K.K., Kim B.K., Hong S.G., Oh H.-M. (2017). Genome sequence of *Deinococcus marmoris* PAMC 26562 isolated from Antarctic lichen. Genome Announc..

[133] Yang J.A., Hong S.G., Oh H.-M. (2017). Genome sequence of *Caballeronia sordidicola* strain PAMC 26510 isolated from *Psoroma* sp., an Antarctic lichen. Korean J. Microbiol..

[134] Biosca E.G., Flores R., Santander R.D., Díez-Gil J.L., Barreno E. (2016). Innovative approaches using lichen enriched media to improve isolation and culturability of lichen associated bacteria. PLoS ONE.

[135] Cernava T., Berg G., Grube M. (2016). High Life expectancy of bacteria on lichens. Microb. Ecol..

[136] Park C.H., Kim K.M., Kim O.-S., Jeong G., Hong S.G. (2016). Bacterial communities in Antarctic lichens. Antarct. Sci..

[137] Sigurbjörnsdóttir M.A., Vilhelmsson O. (2016). Selective isolation of potentially phosphate-mobilizing, biosurfactant-producing and biodegradative bacteria associated with a sub-Arctic, terricolous lichen, *Peltigera membranacea*. FEMS Microbiol. Ecol..

[138] Wedin M., Maier S., Fernandez-Brime S., Cronholm B., Westberg M., Grube M. (2016). Microbiome change by symbiotic invasion in lichens. Environ. Microbiol..

[139] Garg N., Zeng Y., Edlund A., Melnik A.V., Sanchez L.M., Mohimani H., Gurevich A., Miao V., Schiffler S., Lim Y.W. (2016). Spatial molecular architecture of the microbial community of a *Peltigera* lichen. mSystems.

[140] Swamy C.T., Gayathri D., Devaraja T.N., Bandekar M., D’Souza S.E., Meena R.M., Ramaiah N. (2016). Plant growth promoting potential and phylogenetic characteristics of a lichenized nitrogen fixing bacterium, *Enterobacter cloacae*. J. Basic Microbiol..

[141] Han S.-R., Yu S.-C., Ahn D.-H., Park H., Oh T.-J. (2016). Complete genome sequence of *Burkholderia* sp. strain PAMC28687, a potential octopine-utilizing bacterium isolated from Antarctica lichen. J. Biotechnol..

[142] Parrot D., Legrave N., Intertaglia L., Rouaud I., Legembre P., Grube M., Suzuki M.T., Tomasi S. (2016). Cyaneodimycin, a bioactive compound isolated from the culture of *Streptomyces cyaneofuscatus* associated with *Lichina confinis*. Eur. J. Org. Chem..

[143] Oh T.-J., Han S.-R., Kang S., Park H., Kim A.Y. (2016). Complete genome sequence of the xylan-degrading *Mucilaginibacter* sp. strain PAMC26640 isolated from an Arctic lichen. J. Biotechnol..

[144] Grube M., Cernava T., Soh J., Fuchs S., Aschenbrenner I., Lassek C., Wegner U., Becher D., Riedel K., Sensen C.W. (2015). Exploring functional contexts of symbiotic sustain within lichen-associated bacteria by comparative omics. ISME J..

[145] Cernava T., Aschenbrenner I.A., Grube M., Liebminger S., Berg G. (2015). A novel assay for the detection of bioactive volatiles evaluated by screening of lichen-associated bacteria. Front. Microbiol..

[146] Sigurbjörnsdóttir M.A., Andrésson Ó.S., Vilhelmsson O. (2015). Analysis of the *Peltigera membranacea* metagenome indicates that lichen-associated bacteria are involved in phosphate solubilization. Microbiology.

[147] Erlacher A., Cernava T., Cardinale M., Soh J., Sensen C.W., Grube M., Berg G. (2015). *Rhizobiales* as functional and endosymbiotic members in the lichen symbiosis of *Lobaria pulmonaria* L.. Front. Microbiol..

[148] Cernava T., Müller H., Aschenbrenner I.A., Grube M., Berg G. (2015). Analyzing the antagonistic potential of the lichen microbiome against pathogens by bridging metagenomic with culture studies. Front. Microbiol..

[149] Parrot D., Antony-Babu S., Intertaglia L., Grube M., Tomasi S., Suzuki M.T. (2015). Littoral lichens as a novel source of potentially bioactive Actinobacteria. Sci. Rep..

[150] Jiang Y., Wang X., Li G., Li Q., Liu C., Chen X., Wang L., Li Y., Jiang C. (2015). Diversity and anti-microbial activities of actinomycetes associated with three species of lichens. Am. J. Biosci..

[151] Lee Y.M., Kim E.H., Lee H.K., Hong S.G. (2014). Biodiversity and physiological characteristics of Antarctic and Arctic lichens-associated bacteria. World J. Microbiol. Biotechnol..

[152] Aschenbrenner I.A., Cardinale M., Berg G., Grube M. (2014). Microbial cargo: Do bacteria on symbiotic propagules reinforce the microbiome of lichens?. Environ. Microbiol..

[153] Sigurbjörnsdóttir M.A., Heiðmarsson S., Jónsdóttir A.R., Vilhelmsson O. (2014). Novel bacteria associated with Arctic seashore lichens have potential roles in nutrient scavenging. Can. J. Microbiol..

[154] Maier S., Schmidt T.S.B., Zheng L., Peer T., Wagner V., Grube M. (2014). Analyses of dryland biological soil crusts highlight lichens as an important regulator of microbial communities. Biodivers. Conserv..

[155] Anderson O.R. (2014). Microbial communities associated with tree bark foliose lichens: A perspective on their microecology. J. Eukaryot. Microbiol..

[156] Ramírez-Fernández L., Zúñiga C., Carú M., Orlando J. (2014). Environmental context shapes the bacterial community structure associated to *Peltigera cyanolichens* growing in Tierra del Fuego, Chile. World J. Microbiol. Biotechnol..

[157] Kim M.-K., Oh T.-J., Park H. (2014). Antibacterial and antioxidant capacity of polar microorganisms isolated from Arctic lichen *Ochrolechia* sp.. Pol. J. Microbiol..

[158] Kim M.-K., Park H., Oh T.-J. (2014). Antibacterial and antioxidant potential of polar microorganisms isolated from Antarctic lichen *Psoroma* sp.. Afr. J. Microbiol. Res..

[159] Romanovskaia V.A., Parfenova V.V., Bel’kova N.L., Sukhanova E.V., Gladka G.V., Tashireva A.A. (2014). Phylogenetic analysis of bacteria of extreme ecosystems. Mikrobiol. Zh..

[160] Honegger R., Axe L., Edwards D. (2013). Bacterial epibionts and endolichenic actinobacteria and fungi in the Lower Devonian lichen *Chlorolichenomycites salopensis*. Fungal Biol..

[161] Kim M.-K., Oh T.-J., Park H. (2013). Antimicrobial properties of the bacterial associates of the Arctic lichen *Stereocaulon* sp.. Afr. J. Microbiol. Res..

[162] Muggia L., Klug B., Berg G., Grube M. (2013). Localization of bacteria in lichens from Alpine soil crusts by fluorescence in situ hybridization. Appl. Soil Ecol..

[163] Lee D.-H., Hur J.S., Kahng H.-Y. (2013). *Sphingobacterium cladoniae* sp. nov., isolated from lichen, *Cladonia* sp., and emended description of *Sphingobacterium siyangense*. Int. J. Syst. Evol. Microbiol..

[164] Esposito A., Ciccazzo S., Borruso L., Zerbe S., Daffonchio D., Brusetti L. (2013). A Three-scale analysis of bacterial communities involved in rocks colonization and soil formation in high mountain environments. Curr. Microbiol..

[165] Cardinale M., Grube M., Castro J.V., Müller H., Berg G. (2012). Bacterial taxa associated with the lung lichen *Lobaria pulmonaria* are differentially shaped by geography and habitat. FEMS Microbiol. Lett..

[166] Hodkinson B.P., Gottel N.R., Schadt C.W., Lutzoni F. (2012). Photoautotrophic symbiont and geography are major factors affecting highly structured and diverse bacterial communities in the lichen microbiome. Environ. Microbiol..

[167] Cardinale M., Steinová J., Rabensteiner J., Berg G., Grube M. (2012). Age, sun and substrate: Triggers of bacterial communities in lichens. Environ. Microbiol. Rep..

[168] Lee H., Shin Seung C., Lee J., Kim Su J., Kim B.-K., Hong Soon G., Kim Eun H., Park H. (2012). Genome sequence of *Sphingomonas* sp. strain PAMC 26621, an Arctic-lichen-associated bacterium isolated from a *Cetraria* sp.. J. Bacteriol..

[169] Printzen C., Fernández-Mendoza F., Muggia L., Berg G., Grube M. (2012). Alphaproteobacterial communities in geographically distant populations of the lichen *Cetraria aculeata*. FEMS Microbiol. Ecol..

[170] Grube M., Köberl M., Lackner S., Berg C., Berg G. (2012). Host–parasite interaction and microbiome response: Effects of fungal infections on the bacterial community of the Alpine lichen *Solorina crocea*. FEMS Microbiol. Ecol..

[171] Kim M.-K., Park H., Oh T.-J. (2012). Antibacterial properties associated with microorganisms isolated from Arctic lichens. Microbiol. Biotechnol. Lett..

[172] Kim M.-K., Oh T.-J., Park H. (2012). Antioxidant activities of bacterial culture extracts isolated from Arctic lichens. Korean J. Microbiol. Biotechnol..

[173] He Y., Zhang Z. (2012). Diversity of organism in the *Usnea longissima* lichen. Afr. J. Microbiol. Res..

[174] Hamada M., Yamamura H., Komukai C., Tamura T., Suzuki K.-I., Hayakawa M. (2012). *Luteimicrobium album* sp. nov., a novel actinobacterium isolated from a lichen collected in Japan, and emended description of the genus *Luteimicrobium*. J. Antibiot..

[175] Park C.H. (2012). Phylogeny of *Cladonia* in Polar Areas and Microbial Communities in Antarctic Lichens. Ph.D. Thesis.

[176] Bates S.T., Cropsey G.W.G., Caporaso J.G., Knight R., Fierer N. (2011). Bacterial communities associated with the lichen symbiosis. Appl. Environ. Microbiol..

[177] Bjelland T., Grube M., Hoem S., Jorgensen S.L., Daae F.L., Thorseth I.H., Øvreås L. (2011). Microbial metacommunities in the lichen–rock habitat. Environ. Microbiol. Rep..

[178] Schneider T., Schmid E., de Castro J.V., Cardinale M., Eberl L., Grube M., Berg G., Riedel K. (2011). Structure and function of the symbiosis partners of the lung lichen (*Lobaria pulmonaria* L. Hoffm.) analyzed by metaproteomics. Proteomics.

[179] Mushegian A.A., Peterson C.N., Baker C.C.M., Pringle A. (2011). Bacterial diversity across individual lichens. Appl. Environ. Microbiol..

[180] Yamamura H., Ashizawa H., Nakagawa Y., Hamada M., Ishida Y., Otoguro M., Tamura T., Hayakawa M. (2011). Actinomycetospora iriomotensis sp. nov., a novel actinomycete isolated from a lichen sample. J. Antibiot..

[181] Cardinale M., Grube M., Berg G. (2011). *Frondihabitans cladoniiphilus* sp. nov., an actinobacterium of the family *Microbacteriaceae* isolated from lichen, and emended description of the genus *Frondihabitans*. Int. J. Syst. Evol. Microbiol..

[182] Hodkinson B.P. (2011). A Phylogenetic, Ecological, and Functional Characterization of Non-Photoautotrophic Bacteria in the Lichen Microbiome. Ph.D. Thesis.

[183] Da Silva N.M.V., Pereira Filho T., Sa M.T. (2011). Taxonomic characterization and antimicrobial activity of actinomycetes associated with foliose lichens from the Amazonian ecosystems. Aust. J. Basic. Appl. Sci..

[184] Yamamura H., Ashizawa H., Nakagawa Y., Hamada M., Ishida Y., Otoguro M., Tamura T., Hayakawa M. (2011). *Actinomycetospora rishiriensis* sp. nov., isolated from a lichen. Int. J. Syst. Evol. Microbiol..

[185] Lee Y.M., Kim E.H., Hong S.G. (2011). Diversity of Culturable Microorganisms Associated with Antarctic and Arctic Lichens.

[186] Selbmann L., Zucconi L., Ruisi S., Grube M., Cardinale M., Onofri S. (2010). Culturable bacteria associated with Antarctic lichens: Affiliation and psychrotolerance. Polar Biol..

[187] Männistö M.K., Tiirola M., McConnell J., Häggblom M.M. (2010). *Mucilaginibacter frigoritolerans* sp. nov., *Mucilaginibacter lappiensis* sp. nov. and *Mucilaginibacter mallensis* sp. nov., isolated from soil and lichen samples. Int. J. Syst. Evol. Microbiol..

[188] Hodkinson B.P., Lutzoni F. (2009). A microbiotic survey of lichen-associated bacteria reveals a new lineage from the Rhizobiales. Symbiosis.

[189] Grube M., Cardinale M., de Castro J.V., Müller H., Berg G. (2009). Species-specific structural and functional diversity of bacterial communities in lichen symbioses. ISME J..

[190] An S.-Y., Xiao T., Yokota A. (2009). *Leifsonia lichenia* sp. nov., isolated from lichen in Japan. J. Gen. Appl. Microbiol..

[191] Cardinale M., Vieira de Castro J., Müller H., Berg G., Grube M. (2008). In situ analysis of the bacterial community associated with the reindeer lichen *Cladonia arbuscula* reveals predominance of *Alphaproteobacteria*. FEMS Microbiol. Ecol..

[192] Li B., Xie C.-H., Yokota A. (2007). *Nocardioides exalbidus* sp. nov., a novel actinomycete isolated from lichen in Izu-Oshima Island, Japan. Actinomycetologica.

[193] Liba C.M., Ferrara F.I.S., Manfio G.P., Fantinatti-Garboggini F., Albuquerque R.C., Pavan C., Ramos P.L., Moreira-Filho C.A., Barbosa H.R. (2006). Nitrogen-fixing chemo-organotrophic bacteria isolated from cyanobacteria-deprived lichens and their ability to solubilize phosphate and to release amino acids and phytohormones. J. Appl. Microbiol..

[194] Männistö M.K., Häggblom M.M. (2006). Characterization of psychrotolerant heterotrophic bacteria from Finnish Lapland. Syst. Appl. Microbiol..

[195] González I., Ayuso-Sacido A., Anderson A., Genilloud O. (2005). Actinomycetes isolated from lichens: Evaluation of their diversity and detection of biosynthetic gene sequences. FEMS Microbiol. Ecol..

[196] Blanch M., Blanco Y., Fontaniella B., Legaz M.-E., Vicente C. (2001). Production of phenolics by immobilized cells of the lichen *Pseudevernia furfuracea*: The role of epiphytic bacteria. Int. Microbiol..

[197] Scott G.D. (1956). Further Investigation of Some Lichens for Fixation of Nitrogen. New Phytol..

[198] Cardinale M., Puglia A.M., Grube M. (2005). Analysis of the Bacterial Community Associated with Different Species of Lichens: Preliminary Results.

[199] Oren A., Garrity G.M. (2021). Valid publication of the names of forty-two phyla of prokaryotes. Int. J. Syst. Evol. Microbiol..

[200] Pankratov T.A., Kachalkin A.V., Korchikov E.S., Dobrovol’skaya T.G. (2017). Microbial communities of lichens. Microbiology.

[201] Chhipa H., Kaushik N. (2017). Fungal and bacterial diversity isolated from *Aquilaria malaccensis* tree and soil, induces agarospirol formation within 3 months after artificial infection. Front. Microbiol..

[202] Kumar S., Kaushik N., Edrada-Ebel R., Ebel R., Proksch P. (2011). Isolation, characterization, and bioactivity of endophytic fungi of Tylophora indica. World J. Microbiol. Biotechnol..

[203] Masumoto H., Degawa Y. (2019). The effect of surface sterilization and the type of sterilizer on the genus composition of lichen-inhabiting fungi with notes on some frequently isolated genera. Mycoscience.

[204] Yang J.H., Oh S.-Y., Kim W., Woo J.-J., Kim H., Hur J.-S. (2021). Effect of isolation conditions on Diversity of endolichenic fungal communities from a foliose lichen, *Parmotrema tinctorum*. J. Fungi.

